# Supplementary material for: HIV testing and its associated factors among female sex workers in Iran in 2020: Finding from a respondent-driven sampling survey
Source: PLoS One. 2023 Aug 1;18(8):e0289307. doi: 10.1371/journal.pone.0289307 (PMC10393135; doi:10.1371/journal.pone.0289307)
Supplement: Attachment — Submitted filename: Response to Reviewers.docx [file pone.0289307.s003.docx]

PLOS ONE

RE: Response to Reviewers’ comments on Manuscript ID PONE-D-23-01726R1

HIV testing and its associated factors among Female Sex Workers in Iran in 2020: finding from a respondent-driven sampling survey

Dear Dr. Orvalho Augusto

We greatly appreciate the opportunity to revise and resubmit our manuscript entitled “HIV testing and its associated factors among Female Sex Workers in Iran in 2020: finding from a respondent-driven sampling survey”. We found the editor’s and reviewers’ comments helpful and constructive and have tried our best to address all of them. Below is a point-by-point explanation of how we addressed the reviewer's concerns.

We hope that this response will be viewed favorably, and our revised manuscript will be considered for publication in *PLOS ONE.*

Sincerely,

Hamid Sharifi, PhD

Professor in Epidemiology

Journal Requirements:

Please review your reference list to ensure that it is complete and correct. If you have cited papers that have been retracted, please include the rationale for doing so in the manuscript text, or remove these references and replace them with relevant current references. Any changes to the reference list should be mentioned in the rebuttal letter that accompanies your revised manuscript. If you need to cite a retracted article, indicate the article’s retracted status in the References list and also include a citation and full reference for the retraction notice.

Response: Thanks. According to the editor’s comment, we added two related citations for software that we used in the references list.

Additional Editor Comments (if provided):

This manuscript has improved since the last revision. However, some shortcomings persist.

1. The "study design and setting" now has good study design details. It lacks the study settings elements.

Response: Thanks, we checked this note and made the necessary changes. We added subheadings into the study design and setting section for clarification. We, also, added a sentence about study sampling. It reads: “The cities were selected according to the maximum cultural and geographical variation by the study investigators following consultations with the Ministry of Health and to represent different regions across the country”.

2. In the statistical section, please cite the software used (Stata and RDS-Analyst).

Response: Thanks for your comment. We added related citations.

3. For tables 2 and 3, please indicate in the caption whether these are weighted or unweighted analyses.

Response: Thanks, we added this note in the caption in both tables.

4. Figure 1, is it possible to show both weighted and unweighted prevalence?

Response: Thanks for your suggestion. We calculated both of weighted and unweighted prevalence. Also, we replaced weighted prevalence for HIV testing in cities in whole of the text.

Reviewers' comments:

Reviewer's Responses to Questions

**Comments to the Author**

1. If the authors have adequately addressed your comments raised in a previous round of review and you feel that this manuscript is now acceptable for publication, you may indicate that here to bypass the “Comments to the Author” section, enter your conflict of interest statement in the “Confidential to Editor” section, and submit your "Accept" recommendation.

Reviewer #1: All comments have been addressed

Reviewer #2: All comments have been addressed

2. Is the manuscript technically sound, and do the data support the conclusions?

The manuscript must describe a technically sound piece of scientific research with data that supports the conclusions. Experiments must have been conducted rigorously, with appropriate controls, replication, and sample sizes. The conclusions must be drawn appropriately based on the data presented.

Reviewer #1: Yes

Reviewer #2: Yes

3. Has the statistical analysis been performed appropriately and rigorously?

Reviewer #1: Yes

Reviewer #2: Yes

4. Have the authors made all data underlying the findings in their manuscript fully available?

The [PLOS Data policy](http://www.plosone.org/static/policies.action#sharing) requires authors to make all data underlying the findings described in their manuscript fully available without restriction, with rare exception (please refer to the Data Availability Statement in the manuscript PDF file). The data should be provided as part of the manuscript or its supporting information, or deposited to a public repository. For example, in addition to summary statistics, the data points behind means, medians and variance measures should be available. If there are restrictions on publicly sharing data—e.g. participant privacy or use of data from a third party—those must be specified.

Reviewer #1: No

Reviewer #2: Yes

5. Is the manuscript presented in an intelligible fashion and written in standard English?

PLOS ONE does not copyedit accepted manuscripts, so the language in submitted articles must be clear, correct, and unambiguous. Any typographical or grammatical errors should be corrected at revision, so please note any specific errors here.

Reviewer #1: No

Reviewer #2: Yes

6. Review Comments to the Author

Please use the space provided to explain your answers to the questions above. You may also include additional comments for the author, including concerns about dual publication, research ethics, or publication ethics. (Please upload your review as an attachment if it exceeds 20,000 characters)

Reviewer #1: The authors seem to have addressed my comments. Overall, they added clarity to their methodological and statistical approach, which, together with additional elaboration on some points in the Discussion section increased the quality of the manuscript in my opinion. However, there are still some language unclarities in the Abstract e.g. L20 “(RDS) about”, L35 “the odds …was more” and throughout the text, so I suggest them to revise the manuscript carefully for such details and correct as appropriate.

Response: Thanks for your note. We edited the abstract and the manuscript based on your suggestion. For example: “RDS about” changed to “RDS for”.

Reviewer #2: I would like to commend the authors for addressing all of my and the other reviewer's comments. The two sets of age and income variables now in the tables are confusing. I suggest using them as numerical variables only.

Response: Thanks for your comment. We omitted the categorical variables of age and income.

Some minor language notes:
Describe odds as 'higher' or 'lower', not 'more or 'less'.
Please add any abbreiviations to the table footnotes (I see MMT, perhaps there are others).

Response: Thanks. We corrected them.

7. PLOS authors have the option to publish the peer review history of their article ([what does this mean?](https://journals.plos.org/plosone/s/editorial-and-peer-review-process#loc-peer-review-history)). If published, this will include your full peer review and any attached files.
If you choose “no”, your identity will remain anonymous but your review may still be made public.
**Do you want your identity to be public for this peer review?** For information about this choice, including consent withdrawal, please see our [Privacy Policy](https://www.plos.org/privacy-policy).

Reviewer #1: No

Reviewer #2: No

[NOTE: If reviewer comments were submitted as an attachment file, they will be attached to this email and accessible via the submission site. Please log into your account, locate the manuscript record, and check for the action link "View Attachments". If this link does not appear, there are no attachment files.]

While revising your submission, please upload your figure files to the Preflight Analysis and Conversion Engine (PACE) digital diagnostic tool, <https://pacev2.apexcovantage.com/>. PACE helps ensure that figures meet PLOS requirements. To use PACE, you must first register as a user. Registration is free. Then, login and navigate to the UPLOAD tab, where you will find detailed instructions on how to use the tool. If you encounter any issues or have any questions when using PACE, please email PLOS at [figures@plos.org](mailto:figures@plos.org). Please note that Supporting Information files do not need this step.
